# Myeloid-specific deletion of NOX2 prevents the metabolic and neurologic consequences of high fat diet

**DOI:** 10.1371/journal.pone.0181500

**Published:** 2017-08-03

**Authors:** Jennifer K. Pepping, Bolormaa Vandanmagsar, Sun-Ok Fernandez-Kim, Jingying Zhang, Randall L. Mynatt, Annadora J. Bruce-Keller

**Affiliations:** Pennington Biomedical Research Center, Louisiana State University System, Baton Rouge, LA, United States of America; Hospital Infantil Universitario Nino Jesus, SPAIN

## Abstract

High fat diet-induced obesity is associated with inflammatory and oxidative signaling in macrophages that likely participates in metabolic and physiologic impairment. One key factor that could drive pathologic changes in macrophages is the pro-inflammatory, pro-oxidant enzyme NADPH oxidase. However, NADPH oxidase is a pleiotropic enzyme with both pathologic and physiologic functions, ruling out indiscriminant NADPH oxidase inhibition as a viable therapy. To determine if targeted inhibition of monocyte/macrophage NADPH oxidase could mitigate obesity pathology, we generated mice that lack the NADPH oxidase catalytic subunit NOX2 in myeloid lineage cells. C57Bl/6 control (NOX2-FL) and myeloid-deficient NOX2 (mNOX2-KO) mice were given high fat diet for 16 weeks, and subject to comprehensive metabolic, behavioral, and biochemical analyses. Data show that mNOX2-KO mice had lower body weight, delayed adiposity, attenuated visceral inflammation, and decreased macrophage infiltration and cell injury in visceral adipose relative to control NOX2-FL mice. Moreover, the effects of high fat diet on glucose regulation and circulating lipids were attenuated in mNOX2-KO mice. Finally, memory was impaired and markers of brain injury increased in NOX2-FL, but not mNOX2-KO mice. Collectively, these data indicate that NOX2 signaling in macrophages participates in the pathogenesis of obesity, and reinforce a key role for macrophage inflammation in diet-induced metabolic and neurologic decline. Development of macrophage/immune-specific NOX-based therapies could thus potentially be used to preserve metabolic and neurologic function in the context of obesity.

## Introduction

High fat diets and diet-induced obesity are major drivers of metabolic syndrome, which increase the risk for type 2 diabetes, cardiovascular disease, stroke, and cancer (reviewed in [1]). While the pathogenesis of metabolic syndrome is not fully understood, obesity is tightly associated with chronic inflammation in adipose tissue, from which cytokines, acute-phase reactants, and other inflammatory mediators may signal to elicit systemic inflammation [2], [3]. For example, infiltration of macrophages into adipose tissue is well established following high-fat feeding [4], and has been linked to increased inflammation as well as dyslipidemia and insulin resistance [4]. Furthermore, diet-induced obesity is associated with brain pathology, neurobehavioral dysfunction, and dementia (reviewed in [5]), which is likewise linked to systemic inflammation [6]. In light of the increasing prevalence of obesity and metabolic syndrome [7], novel therapies that effectively block or attenuate adipose and/or systemic inflammation could lead to important gains in public health.

While the molecular link(s) between adiposity and systemic inflammation remain incompletely understood, activation of the pro-inflammatory/pro-oxidant enzyme NADPH oxidase is likely to participate. NADPH oxidase is a multi-subunit superoxide-producing complex widely considered to be a major source of reactive oxygen species produced in phagocytic cells; and has been implicated in various pathological conditions, including cardiovascular disease [8], cancer [9], stroke [10], and neurodegenerative disorders such as Alzheimer’s and Parkinson’s disease [11],[12]. With regard to obesity, studies show that NADPH oxidase activity/expression is increased in laboratory animals given high fat diet [13], [14], and further indicate that diet-induced NADPH oxidase activity mediates obesity-related cytokine/chemokine release as well as insulin resistance, hyperlipidemia, and liver steatosis [14]. Finally, mice deficient in NOX2 have been shown to resist some metabolic effects of high fat diets, with attenuated adipose pathology, preserved adipose function, and improved glucose tolerance [15].

While these data suggest a direct role for NADPH oxidase in obesity-related inflammation, NADPH oxidase also plays important roles in *physiologic* processes [16],[17], and well-established adverse effects of systemic NOX2 deletion on cognitive function [17] rule out indiscriminate NADPH oxidase inhibition as a viable therapy for obesity. To determine if targeted NADPH oxidase deletion could be both safe and effective, we generated mice with a floxed allele of the NAPDH oxidase subunit NOX2 and crossed these with mice expressing Lyz2-Cre to generate mice lacking NOX2 in all myeloid lineage cells, including monocytes, macrophages, and microglia. To then reveal the exact role of myeloid NADPH oxidase in the pathophysiology of obesity, C57Bl/6 (NOX2-FL) and myeloid-deficient NOX2 (mNOX2-KO) mice were given high fat diet for 16 weeks, and subject to comprehensive metabolic, behavioral, and biochemical analyses.

## Materials and methods

### Transgenic animal generation and treatment

This study was carried out in strict accordance with PHS/NIH guidelines on the use of experimental animals, and the Institutional Animal Care and Use Committee at the Pennington Biomedical Research Center approved all experimental protocols. Cybb conditional targeting vector was generated by the Knockout Mice Project (KOMP Project ID CSD45129), where exon 5 of Cybb gene was flanked by two directional loxp sites, and electroporated into C57BL/6N-PRX-B6N mouse embryonic stem cells (Jax cat # 012448C01). Verified clones were injected into albino B6 blastocysts (C57BL/6J-Tyrc-^2J^ Jax Stock Number 000058), and resulting chimeras crossed with albino B6 females for germline transmission. The Frt flanked region (containing the selectable marker and reporter gene) was removed by crossing Cybb ^fl/+^ with ACTB:FLPe B6J mice (Jax Stock No: 005703). Germ-line transmission and the establishment of homozygous NOX2 floxed mice (NOX2-FL) were confirmed by allele-specific genotyping analysis of PCR products.

Female NOX2-FL were crossed with male Lyz2-Cre transgenic mice (Jackson Laboratory, Bar Harbor, ME), to generate male F1 progeny lacking NOX2 specifically in myeloid lineage cells (mNOX2-KO). While mice with body-wide loss of NOX2 are a well-established model of chronic granulomatous disease and must be housed under sterile conditions to accommodate their phenotype [18], none of the conditional mNOX2-KO mice showed any signs of granuloma formation, even when group-housed under standard (i.e., non-sterile) conditions for up to 24 months. Male mNOX2-KO and NOX2-FL mice were group-housed with genotype-matched littermates in standard caging with a 12:12 light:dark cycle and ad libitum access to food and water. Mice were given either high fat diet (HFD: 60% fat, D12492 from Research Diets (New Brunswick, NJ)) or low fat control diet (CD: 10% fat, D12450B) for 16 weeks, and data were compiled from 2 separate experiments to total 18–20 animals in each group.

Body weight and body composition (measured using a Bruker minispec LF90 time domain Nuclear Magnetic Resonance (NMR) analyzer, Bruker Optics, Billerica MA) were measured twice monthly, while food intake was measured weekly. Daily energy intake per mouse was estimated by multiplying diet caloric density (3.85 for CD; 5.24 for HFD) by average grams of feed consumed per cage/#mice/day). All mice were humanely euthanatized via isoflurane inhalation and cardiac puncture after a brief (6 hr) fast. Peritoneal cells were collected by lavage with sterile PBS, and adipose deposits/internal organs were collected and weighed, and then assessed immediately, frozen and stored at -80°C, or fixed in 10% neutral buffered formalin.

### Clinical chemistry

Fasting blood glucose was measured in tail blood using a glucometer (Ascensia Elite, Bayer, Mishawaka, IN) immediately before euthanasia. Whole blood was collected into EDTA-coated tubes and centrifuged. Levels of total cholesterol, HDL cholesterol, LDL cholesterol, and triglycerides in plasma were measured colorimetrically (Wako Chemicals, Richmond, VA), while insulin was evaluated by ELISA (Crystal Chem Inc., Downers Grove IL).

### Flow cytometry and cell sorting

To confirm NOX2 deletion in myeloid-lineage cells, peritoneal cells were isolated from 5 month-old male mNOX2-KO and NOX2-FL mice by lavage with sterile PBS. Cells (1 x 10^6^ cells/ml) were washed, stained with PE-conjugated antibodies to F4/80 (eBiosciences), and F4/80^+^ monocytes/macrophages were isolated using BD FACS Aria II (San Jose, CA). Total RNA extraction from sorted cells was carried out using RNeasy Micro Kit (Qiagen) and DNAse digestion was performed on the RNeasy spin column to remove potential genomic DNA contamination in total RNA. Cybb gene expression in F4/80^+^ cells was determined by RT-PCR using mouse cyclophillin B as an internal standard for normalization of gene expression as described previously [19].

To evaluate immune cell populations in adipose tissue, visceral epididymal fat pads were subject to collagenase digestion and filtration to isolate stromalvascular cells (SVC) [20]. SVC were stained with antibodies to F4/80, CD3, Ly6G, and Ly6C (eBiosciences and Biolegend) to identify macrophage, T lymphocyte, neutrophil and monocyte cells, respectively. FACS analyses were conducted using FACS Calibur (BD Pharmingen) with data analyzed by post collection compensation using FlowJO (Treestar Inc) software.

### Quantitative real-time PCR

Gene expression in cells isolated from peritoneal lavage was determined by quantitative real-time PCR in the ABI-Prism 7900 HT Sequence Detection system (Applied Biosystems, Foster City, California) using 18s as an internal standard as described previously by our laboratory [21]. Each cDNA sample was assayed in triplicate, and the cycle number at first detection of signal above threshold (Ct) was determined. Analysis was performed with ABI Prism 7000 SDS Software Version 1.0 (Applied Biosystems). Triplicate measurements for a given sample were averaged, and the values for each target gene were then normalized to measurements for *18s* cDNA in the same sample using the formula: Ct_target gene_−Ct_18s_ = ΔCt. Group comparisons (HFD vs CD for each genotype, HFD in NOX2-FL vs HFD in mNOX2-KO) were based on: ΔΔCt = ΔCt_grp1_ – ΔCt_grp2_, and fold-changes in gene expression were calculated from ΔΔCt using the following formula (ABS(ΔΔCt)/ ΔΔCt) x 2^(ABS(ΔΔCt))^.

### Histological analyses of adipose tissue

Epididymal adipose depots were drop-fixed in 10% neutral buffered formalin and processed for paraffin embedding. Adipocyte size in 5 μm sections was measured by an investigator blinded to the experimental grouping in 20X microscope fields dividing the area of predefined grids by the total number of adipocytes within as described previously [15]. For analyses of macrophage crowns, tissue sections were processed using anti-Iba-1 (1:100, Wako Chemicals, Richmond, VA), biotinylated-linked secondary antibodies, and visualized using diaminobenzidine (Vector Laboratories, Burlingame, CA). Crown density was obtained by counting the total number of crown-like structures in 10X microscope fields from each section. For all histological analyses, 3 replicate tissue sections were analyzed, with 5 fields counted in each section, for a total of 15 fields averaged per sample.

### Western blot of adipose and brain tissue

Adipose tissue samples were homogenized in RIPA buffer (G biosciences, St. Louis, MO), cleared by centrifugation, processed for chemiluminescence-based detection using anti-GADD153/CHOP (Abcam PLC), anti-GRP78 (Novus Biologicals LLC), and anti-total STAT5 (Abcam PLC). Blots for brain tissues were probed using anti-MMP2 (Abcam Inc.), anti-MMP-9 (Abcam Inc.), anti-iNOS (Abcam Inc.), anti-COX2 (Abcam Inc.), anti-synapsin 1 (Thermo Fisher Scientific, Pittsburg, PA), anti-phospho(S553)-synapsin 1 (Abcam Inc.), anti-synapse associated protein 97 (Abcam Inc.), and anti-tubulin (Wako Chemicals USA Inc.). To ensure accurate quantification across multiple blots, samples from all groups (HFD and CD in both NOX2-FL and mNOX2-KO) were included in each individual blot, and data were calculated initially relative to tubulin (internal loading control), and then expression in HFD mice was then calculated/presented as percent expression relative to CD mice of the same genotype as described previously[15].

### Fear conditioning memory task

Each mouse was individually evaluated for fear conditioning using an automated, video-based fear conditioning system (Med-Associates, St. Albans, VT) as described previously [22],[23]. The apparatus consists of a “startle chamber” used on days 1 and 2, which is an 8×15×15-cm acrylic and wire mesh cage located within a custom designed 90×70×70 ventilated sound-attenuating chamber, and the unique context is reinforced with an anise-based scent applied to each cage before testing. Animal movement within the apparatus results in displacement of an accelerometer (model U321AO2; PCB Piezotronics, Depew, NY, USA). Acquisition of fear conditioning on day 1 consists of 5 minutes acclimation to the startle chamber, followed by five consecutive 30 second auditory stimuli (85 db, 4 KHz) co-terminating with a mild footshock (0.5 mA × 1 sec), with 30 second recovery periods between tones. On day 2, mice return to the same chambers, but no stimuli are applied to evaluate freezing responses to context. On day 3, mice are placed in an entirely separate chamber located in a different room to remove all contextual cues, and after 5 minute habituation, a continuous tone (85 db, 4 KHz) is applied for 5 minutes. Freezing behavior is recorded as a measure of memory of the conditioned response to the tone.

### Statistical analyses

All data are presented as mean ± standard error of measurement. Baseline differences in body size and organ weight in NOX2-FL to mNOX2-KO mice were analyzed by 2-tailed, unpaired t-tests. Body weight and composition data, adipocyte size, crowns, and all metabolic data in CD- and HFD-fed mice were analyzed with 2-way analyses of variance (ANOVA), followed by planned Bonferroni post-tests to determine the differential effects of HFD in NOX2-FL as compared to mNOX2-KO mice. Additionally, planned comparisons of NOX2-FL to mNOX2-KO mice under both CD and HFD conditions were carried out using 1-way ANOVA. Protein expression values generated by Western blot (ratios of expression over tubulin) were normalized to percent CD for each genotype and were analyzed by 2-tailed, unpaired t-tests to determine differences between HFD and CD groups within each genotype. Differences in mRNA expression (ΔCt) between groups were also evaluated by 2-tailed, unpaired t-tests. Statistical significance for all analyses was accepted at p < 0.05, and *, **, and *** represent p < 0.05, p < 0.01, and p < 0.001, respectively.

## Results

### Generation and characterization of mNOX2-KO mice

To determine the consequences of cell type-specific NOX2 deletion, newly engineered mice in which LoxP sequences were placed on either side of exon 5 of the NOX2 gene were generated (NOX2-FL) and crossed with Lyz2-Cre transgenic mice to generate mice lacking NOX2 in all myeloid lineage cells (mNOX2-KO). Male progeny from NOX2-FL ^x^ NOX2-FL crosses were used as controls, and there were no differences in pregnancy rates, litter sizes, or sex balance of offspring between NOX2-FL ^x^ NOX2-FL and NOX2-FL ^x^ Lyz2Cre breeding pairs (data not shown). To confirm NOX2 knock-down in myeloid cells, RT-PCR analysis of Cybb gene expression relative to cyclophilin B in F4/80-positive cells was carried out as described in Methods. Data show greater than 95% reduction in relative Cybb mRNA expression in cells isolated from mNOX2-KO compared to NOX2-FL cells (Table 1), confirming the effective loss of NOX2 expression. Comparison of baseline differences in body size between the two groups of male mice revealed that adult (5–7 month old) mNOX2-KO mice were modestly but significantly smaller than NOX2-FL mice, with deceases in body weight and snout-to-anus length (Table 1). There were no group differences in the relative size (mg per gram body weight) of heart, liver, kidney, or thymus, although the mNOX2-KO mice did have relatively larger brains (Table 1). Interestingly, mNOX2-KO mice had significantly larger spleens, as has been reported for global NOX2 knockout mice [24]. To determine if loss of NOX2 from myeloid lineage cells significantly altered leukocyte populations in visceral adipose, epididymal adipose depots were removed from adult male NOX2-FL and mNOX2-KO, and stromovascular cells were isolated and subject to 4-color flow cytometry as described in Methods. Data show that the balance of T cell, monocyte, macrophage, and neutrophil populations within visceral adipose were not significantly different in mNOX2-KO mice as compared to NOX2-FL mice (Table 1). Similar analyses in peripheral blood preparations likewise showed no genotype-based differences in leukocyte cell numbers between mNOX2-KO and NOX2-FL mice (data not shown).

**Table 1:** Baseline characterization of NOX2-FL and mNOX2-KO mice.

	NOX2-FL	mNOX2-KO
Cybb mRNA (relative expression)	3006.32	140.05
Body weight (g)	32.2 ± 1.2	30.0 ± 1.1 ##
Snout-anus length (mm)	101.3 ± 0.6	98.4 ± .05 #
Heart weight (mg/g)	6.2 ± 0.5	6.0 ± 0.5
Liver weight (mg/g)	53.8 ± 2.2	48.6 ± 2.4
Kidney weight (mg/g)	7.0 ± 0.2	6.9 ± 0.2
Brain weight (mg/g)	14.3 ± 0.4	16.2 ± 0.5 #
Thymus weight (mg/g)	1.2 ± 0.1	1.2 ± 0.1
Spleen weight (mg/g)	3.3 ± 0.1	8.2 ± .08 ###
Epididymal fat (mg/g)	11.0 ± 1.0	7.4 ± 0.6 ##
Inguinal fat (mg/g)	4.9 ± 0.5	4.1 ± 0.2
Epididymal adipose leukocytes		
Tcells (%)	57.6 ± 6.4	51.1 ± 5.0
Neutrophils (%)	4.5 ± 0.7	4.2 ± 0.9
Monocytes (%)	39.5 ± 2.3	34.1 ± 3.7
Macrophages (%)	16.6 ± 1.7	16.5 ± 3.0

Macrophage/monocyte Cybb mRNA expression, measures of body size, organ weights (relative to body weight (mg/g)), and visceral adipose leukocyte populations in tissues isolated from untreated, 5–7 month-old male NOX2-FL and mNOX2-KO mice maintained on chow diet were assessed. Cybb mRNA expression is presented as mean expression relative to Cyclophilin B in 2 samples/group (each comprised of isolated F4/80+ peritoneal cells pooled from 2 mice). All other data are mean ± SEM, and were analyzed by 2-tailed, unpaired t-tests. Statistically significant genotype-based differences in mNOX2-KO mice as compared to NOX2-FL mice are noted by

#p<0.05

##p<0.01, and

###p<0.001

### Body weight and composition in NOX2-FL and mNOX2-KO mice following high fat diet

**Fig 1:**
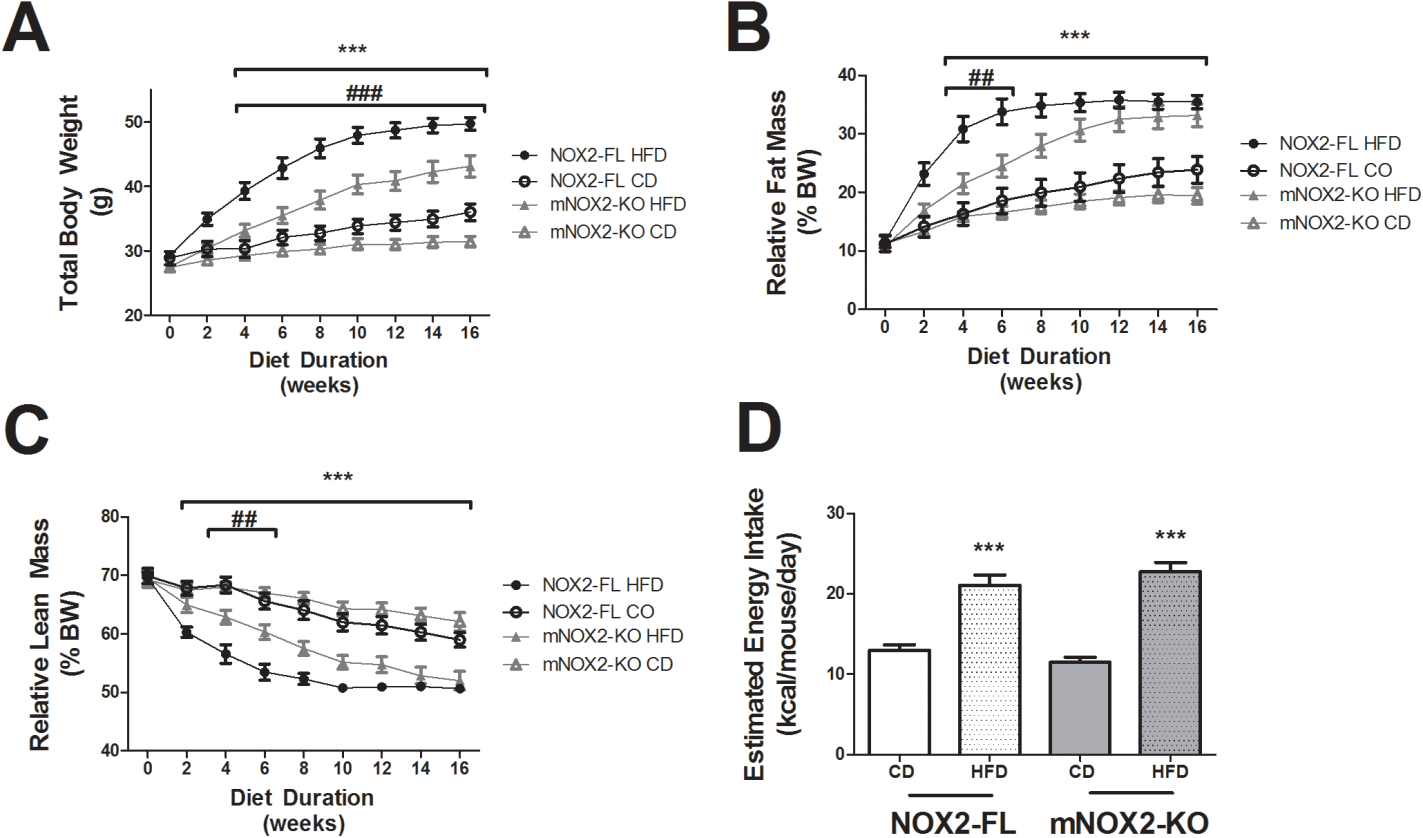
Effects of HFD on body weight, total body fat, and energy intake in NOX2-FL and mNOX2-KO mice. 4-month old male C57Bl/6 (NOX2-FL) mice and myeloid-specific NOX2 knock-out (mNOX2-KO) mice were placed on high fat diet (HFD), or the nutritionally matched low fat control diet (CD) for 16 weeks. **(A)** Trajectory of body weight in NOX2-FL and mNOX2-KO mice. Data are means and SEM, and *** indicates significant (p < 0.001) increases in body weight noted in both NOX2-FL and mNOX2-KO mice given HFD as compared to CD-fed mice; while ### indicates significantly (p < 0.001) decreased body weight in mNOX2-KO mice given HFD as compared to HFD-fed NOX2-FL mice. **(B)** Trajectory of total body fat as a percentage of total body weight. *** indicates significant (p < 0.001) increases in body fat noted in both NOX2-FL and mNOX2-KO mice given HFD as compared to CD-fed mice; while ## indicates significantly (p < 0.01) decreased body fat in mNOX2-KO mice given HFD as compared to HFD-fed NOX2-FL mice after 4 and 6 weeks on HFD. **(C)** Trajectory of total body lean mass as a percentage of total body weight. *** indicates significant (p < 0.001) decreases in relative lean mass noted in both NOX2-FL and mNOX2-KO mice given HFD as compared to CD-fed mice; while ## indicates significantly (p < 0.01) increased lean mass in mNOX2-KO mice given HFD as compared to HFD-fed NOX2-FL mice after 4 and 6 weeks on HFD. **(D)** Average energy intake in NOX2-FL and mNOX2-KO mice was estimated over time following administration of CD or HFD. *** indicates significant (p < 0.001) increase in energy intake in HFD-fed mice as compared to CD-fed mice.

During diet exposure, body weights of mice progressively diverged such that HFD-fed mice weighed significantly more than CD mice after 4 weeks (Fig 1A). Statistically, ANOVA for genotype X diet at the end of exposure revealed a significant main effect of diet on body weight (F_(1,68)_ = 104.3, p < 0.0001). The effect of genotype on body weight was also significant (F_(1,68)_ = 20.02, p = 0. 0001), but the interaction was not. Planned comparisons of mNOX2-KO and NOX2-FL mice showed no significant differences between body weights in mice given CD, and that HFD-fed mNOX2-KO mice weighed significantly less than HFD-fed NOX2-FL mice after 4 weeks of diet (Fig 1A). In addition to body weight, body fat was measured using NMR as described in Methods (Fig 1B). Statistically, ANOVA to measure the effects of genotype and diet on % body fat at the end of diet exposure revealed a significant main effect of diet (F_(1,68)_ = 52.34, p < 0.0001), but no effect genotype and no significant interaction between diet and genotype on body fat. Planned comparisons of mNOX2-KO and NOX2-FL mice revealed no genotype-based differences in % body fat in mice given CD, but did indicate a delay in fat accumulation in mNOX2-KO mice as compared to NOX2-FL during the first 6 weeks of HFD (Fig 1B). Relative body lean mass was likewise measured using NMR (Fig 1C), and ANOVA to measure the effects of genotype and diet on % lean mass showed a significant main effect of diet (F_(1,68)_ = 41.74, p < 0.0001), but no effect of genotype and no interaction. Planned comparisons of mNOX2-KO and NOX2-FL mice revealed no genotype-based differences in % lean mass in mice given CD, but did reveal a delay in the relative loss of lean mass in mNOX2-KO as compared to NOX2-FL mice in the first 6 weeks of HFD (Fig 1C). Analysis of food intake during this period was also conducted, and while data show that high fat-fed mice consumed a greater number of kcal/day than mice given CD, there were no differences between NOX2-FL and mNOX2-KO mice with regards to diet (CD or HFD) ingestion (Fig 1D), indicating the increased body weight in NOX2-FL mice was not caused by increased food intake.

### Visceral adipocyte injury and inflammation in NOX2-FL and mNOX2-KO mice

**Fig 2:**
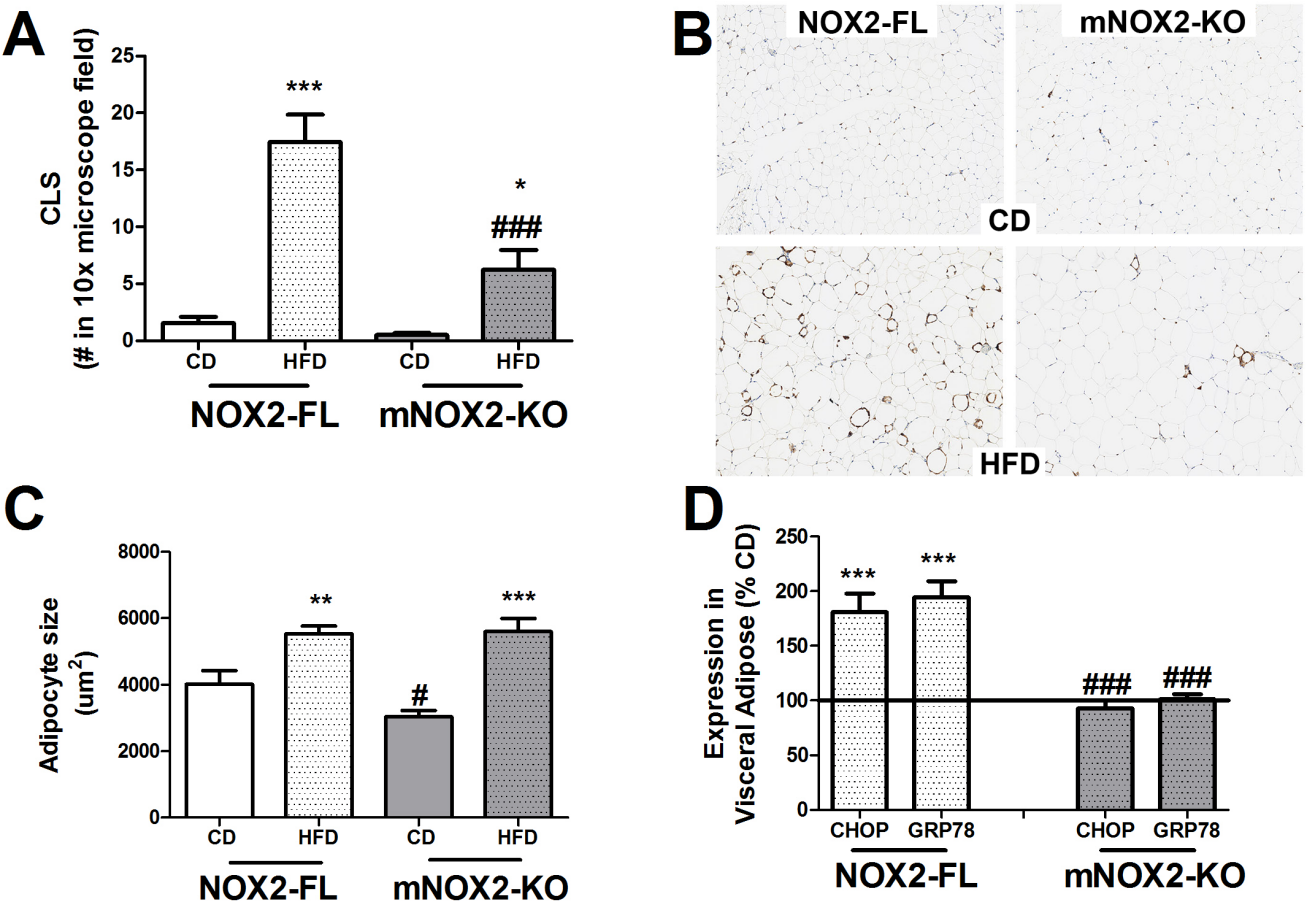
Effects of HFD on adipose macrophage infiltration, hypertrophy, and injury in NOX2-FL and mNOX2-KO mice. Visceral epididymal fat pads were collected at the end of the 16-week feeding trail. **(A)** Blinded quantification of crown-like structures (CLSs) composed of continuous Iba-1-positive macrophages. Data are means and SEM, and *** and * indicates significant (p < 0.001 and p < 0.05) increases in CLS in HFD-fed NOX2-FL and mNOX2-KO mice compared to mice on CD, while ### depicts significant decrease in CLS in HFD-fed mNOX2-KO mice as compared to HFD-fed NOX2-FL mice. **(B)** Representative images of Iba-1 immunostaining in epididymal fat from all groups of mice. **(C)** Epididymal adipocyte size. Data are means and SEM, and ** and *** indicates significant (p < 0.01 and p < 0.0501) increases in adipocyte size in HFD-fed NOX2-FL and mNOX2-KO mice compared to mice on CD. # depicts significant decrease in epididymal adipocyte size in CD-fed mNOX2-KO mice as compared to CD-fed NOX2-FL mice. **(D)** Expression of GADD153/CHOP and GRP78 in tissue homogenates prepared from epididymal adipose depots. Data depict mean ± SEM expression in HFD mice presented as % values in CD mice (100% line on graph). *** indicates significant (p < 0.001) increases in expression in HFD-fed NOX2-FL mice compared to CD-fed NOX2-FL mice, while ### indicates significant (p < 0.001) decreases in expression in HFD-fed mNOX2-KO mice as compared to HFD-fed NOX2-FL mice.

To assess the role of NOX2-positive macrophages in obesity-induced inflammatory and pathogenic changes in adipose, we investigated the impact of conditional NOX2 deletion on macrophage infiltration into visceral adipose tissue by measuring expression of Iba-1[25]. Crown-like structures (CLSs) consisting of Iba-1-positive macrophages in epididymal adipose depots were markedly increased following HFD, particularly in NOX2-FL mice (Fig 2A and 2B). Specifically, ANOVA for the effects of genotype and diet on epididymal CLS revealed a significant main effect of diet (F_(1,66)_ = 50.11, p < 0.0001) and of genotype (F_(1,66)_ = 16.20, p < 0.0001), with a significant interaction (F_(1,66)_ = 11.02, p = 0.0015). Planned comparisons revealed no differences in epididymal CLS number in CD-fed mice, but did show that HFD significantly increased the numbers of CLS in both NOX2-FL and mNOX2-KO mice (Fig 2A). However, the number of CLS in HFD-fed mNOX2-KO mice was significantly less that in HFD-fed NOX2-FL mice (Fig 2A). Representative images of Iba-1 stained tissue sections reveal obvious Iba-1 positive CLS in visceral epididymal adipose depots isolated from HFD-fed NOX2-FL mice, but not from HFD-fed mNOX2-KO mice or mice given CD (Fig 2B).

The size of individual adipocytes within visceral epididymal adipose depots was evaluated, and data show that HFD significantly increased the overall size of visceral adipocytes (Fig 2C). Specifically, ANOVA for the effects of genotype and diet on epididymal adipocyte size revealed a significant main effect of diet (F_(1,66)_ = 38.61, p < 0.0001), but no effect of genotype and no interaction. Planned comparisons revealed that inguinal adipocytes in CD-fed NOX2-FL mice were significantly larger than adipocytes in CD-fed mNOX2-KO (Fig 2C). However, HFD consumption increased visceral adipocyte size similarly in both NOX2-FL and mNOX2-KO mice (Fig 2C). To determine if the hypertrophy and inflammation noted in visceral adipose was accompanied by cell injury, adipocyte injury in visceral adipose of NOX2-FL and mNOX2-KO mice was estimated by evaluating the expression GADD153/CHOP and GRP78, both of which are known to be increased in the context of obesity and thought to reflect inflammation-induced endoplasmic reticulum (ER) stress [26]. Data show that HFD significantly increased GADD153 expression only in NOX2-FL mice (t_(31)_ = 4.55, p < 0.0001; Fig 2D). Likewise, expression of GRP78 was also significantly increased by HFD in NOX2-FL mice (t_(27)_ = 6.70, p < 0.0001), but not in mNOX2-KO mice (Fig 2D), suggesting that although mNOX2-KO adipocytes became hypertrophic, these cells did not appear to be subject to ER stress.

Finally, as HFD consumption has been shown to increase pro-inflammatory, M1-type gene expression in peritoneal [27] macrophages, we investigated the impact of conditional NOX2 deletion on gene expression in peritoneal macrophages using qPCR. Analyses were limited to four well-characterized markers of inflammation (TNF-α, IL-6, CD86, and MHC class II) and one anti-inflammatory, M2 marker (Arginase 1) [28, 29]. Data show that pro-inflammatory gene expression was significantly increased, and arginase 1 expression decreased, by HFD in peritoneal macrophages in NOX2-FL, but not mNOX2-KO mice (Table 2). Furthermore, direct comparison of macrophage gene expression in HFD-fed mice revealed 2–3 fold higher expression of pro-inflammatory genes in HFD-fed NOX2-FL mice compared to HFD-fed mNOX2-KO (Table 2), suggesting that NOX2 participates in inflammatory signaling in cells of the visceral cavity.

**Table 2:** Changes in gene expression in peritoneal cells isolated from NOX2-FL and mNOX2-KO mice.

	NOX2-FL: HFD vs CD	mNOX2-KO: HFD vs CD	HFD: NOX2-FL vs mNOX2-KO
**TNF**	1.55	-1.43	2.08 #
**IL-6**	2.59 *	1.03	3.15 #
**CD86**	1.83 *	-1.31	2.64 ##
**MHCII**	2.01 **	-1.76	2.55 #
**Arginase 1 (M2)**	-3.20 *	-1.28	-1.46

Peritoneal cells were collected from NOX2-FL and mNOX2-KO mice at the end of the 16-week feeding trail and differences in gene expression levels were determined by quantitative real-time PCR. From the ΔΔC_t_-values, changes in the expression of specific transcripts were calculated as described in Methods. Data represent fold change, and indicate significant (changes in gene expression in HFD-fed NOX2-FL mice compared to CD-fed NOX2-FL

*p < 0.05 and

**p < 0.01

while significant increases in gene expression in HFD-fed NOX2-FL mice as compared to HFD-fed mNOX2-KO mice are indicated by

#p < 0.05 and

# # p < 0.01

### Metabolic dysfunction in NOX2-FL and mNOX2-KO mice

**Fig 3:**
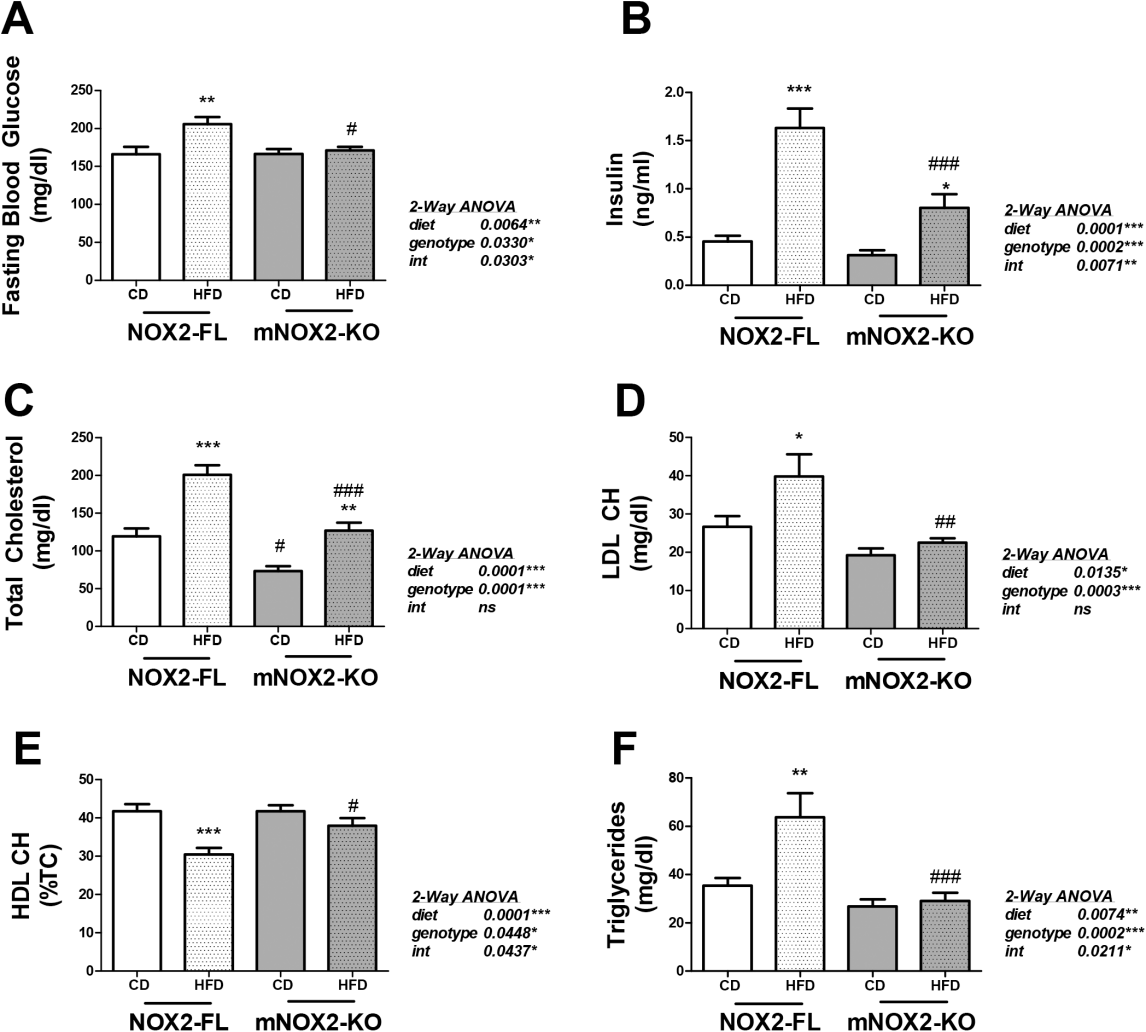
Effects of HFD on glucose regulation and hyperlipidemia in NOX2-FL and mNOX2-KO mice. **(A)** Fasting blood glucose levels. Data are mean and SEM, and ** indicates significant (p < 0.01) increase in fasting blood glucose level in HFD-fed NOX2-FL mice as compared to CD-fed NOX2-FL mice, while # depicts significant decrease in fasting glucose in HFD-fed mNOX2-KO mice as compared to HFD-fed NOX2-FL mice. **(B)** Fasting insulin levels. *** and * indicate significant (p < 0.001 and p < 0.05) increases in fasting insulin in HFD-fed NOX2-FL mice and mNOX2-KO mice compared to CD-fed mice, while ### depicts significant decrease in insulin in HFD-fed mNOX2-KO mice as compared to HFD-fed NOX2-FL mice. **(C)** Total cholesterol levels. Data are means and SEM, and *** and ** indicates significant (p < 0.001 and p < 0.01) increases in total cholesterol in HFD-fed NOX2-FL and mNOX2-KO mice compared to mice on CD. # indicates decreased (p < 0.05) total cholesterol in CD-fed mNOX2-KO mice compared to CD-fed NOX2-FL mice, while ### indicates decreased total cholesterol in HFD-fed mNOX2-KO mice compared to HFD-fed NOX2-FL mice. **(D)** LDL cholesterol, data are means and SEM, and * indicates significant (p < 0.05) increases in LDL cholesterol in HFD-fed NOX2-FL compared to CD-fed NOX2-FL mice, while ## indicates decreased (p < 0.01) LDL cholesterol in HFD-fed mNOX2-KO mice compared to HFD-fed NOX2-FL mice. **(E)** HDL cholesterol depicted as % total cholesterol. Data are mean and SEM, and *** indicates significantly (p < 0.001) decreased HDL cholesterol in HFD-fed NOX2-FL mice as compared to CD-fed NOX2-FL mice, while # depicts significantly increased HDL cholesterol in HFD-fed mNOX2-KO mice as compared to HFD-fed NOX2-FL mice. **(F)** Triglyceride levels, data are mean and SEM, and ** indicates significantly (p < 0.01) increased triglycerides in HFD-fed NOX2-FL mice as compared to CD-fed NOX2-FL mice, while ### depicts significantly decreased (p < 0.001) triglycerides in HFD-fed mNOX2-KO mice as compared to HFD-fed NOX2-FL mice.

To quantify metabolic dysfunction in NOX2-FL and mNOX2-KO mice, data on glucose regulation and hyperlipidemia were collected. Fasting glucose and insulin were measured at the end of diet exposure, and 2-way ANOVA on the effects of genotype and diet on fasting blood glucose revealed significant main effects of diet (F_(1,67)_ = 7.94, p = 0.0064) and genotype (F_(1,67)_ = 4.74, p < 0.0330), with a statistically significant interaction between diet and genotype (F_(1,67)_ = 4.90, p = 0.0303; Fig 3A). Post-hoc tests showed that HFD increased glucose levels in NOX2-FL mice but not in mNOX2-KO mice, while planned comparisons revealed that glucose levels in HFD-fed NOX2-FL mice were significantly higher than levels in HFD-fed mNOX2-KO mice (Fig 3A). Conversely, 2-way ANOVA for fasting insulin revealed a significant effect of diet (F_(1,35)_ = 14.44, p = 0.0014), but no effect of genotype and no interaction (Fig 3B). Post-hoc tests showed that HFD increased insulin levels in both NOX2-FL and mNOX2-KO mice, while planned comparisons revealed much higher insulin levels in HFD-fed NOX2-FL mice compared to HFD-fed mNOX2-KO mice (Fig 3B).

Studies next assessed a panel of bioactive serum lipids in NOX2-FL and mNOX2-KO mice, measured under fasted conditions. Data show a significant effect of diet (F_(1,61)_ = 42.38, p < 0.0001) and of genotype (F_(1,61)_ = 33.45, p < 0.0001) on total cholesterol, but no interaction. Planned comparisons revealed that total cholesterol levels in mNOX2-KO mice were significantly lower than levels in NOX2-FL mice under both CD- and HFD-fed conditions (Fig 3C). Likewise, there were significant effects of diet (F_(1,66)_ = 6.45, p = 0.0135) and genotype (F_(1,66)_ = 14.71, p = 0.0003) on LDL cholesterol, but no interaction (Fig 3D). Additionally, HFD increased LDL cholesterol in NOX2-FL, but not mNOX2-KO mice (Fig 3D). Levels of HDL cholesterol are presented as % total cholesterol, and data show a significant effect of diet (F_(1,66)_ = 17.22, p < 0.0001) and of genotype (F_(1,66)_ = 4.18, p < 0.0448) on HDL, with a significant interaction (F_(1,66)_ = 4.23, p < 0.0437). Planned comparisons revealed that HFD decreased HDL levels only in NOX2-FL mice (Fig 3E). Finally, data show a significant effect of diet (F_(1,67)_ = 7.64, p < 0.0074) and of genotype (F_(1,67)_ = 15.38, p < 0.0002) on circulating triglycerides, with a significant interaction (F_(1,67)_ = 5.58, p < 0.0211). Planned comparisons revealed that HFD increased triglyceride levels only in NOX2-FL mice (Fig 3F).

### Cognitive function and brain injury in NOX2-FL and mNOX2-KO mice

**Fig 4:**
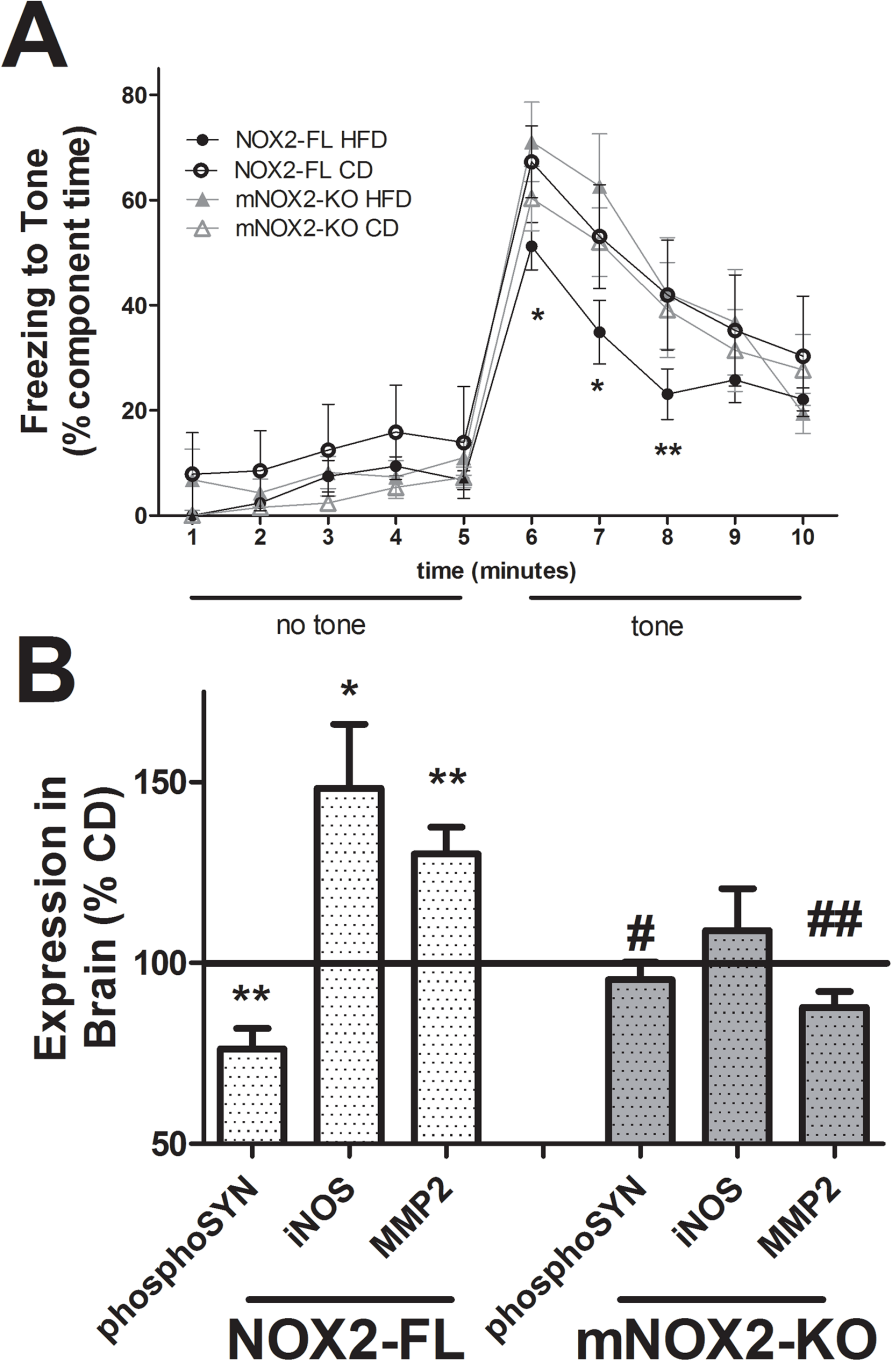
Effects of HFD on markers of brain injury in NOX2-FL and mNOX2-KO mice. **(A)** Memory performance in fear conditioning assay. Data are means ± S.E.M. of composite freezing behavior, and were analyzed by 2-way ANOVA. *and ** indicate significant (p<0.05, p<0.01, respectively) decreases in freezing behavior following the conditioned stimulus (tone cue) in HFD-fed NOX2-FL mice as compared to CD-fed NOX2-FL mice. **(B)** Markers of synaptic density (expression of the phosphorylated form of the pre-synaptic protein synapsin 1) and inflammation (expression of iNOS and MMP2) in anterior cortex. Data depict mean ± SEM expression in HFD mice presented as % values in CD mice (100% line) on graph. * and ** indicate significant (p < 0.05 and 0.01, respectively) changes in expression in NOX2-FL HFD mice compared to NOX2-FL CD mice, while # and ## depicts significant (p < 0.05 and p < 0.01) changes in expression in HFD-fed mNOX2-KO mice as compared to HFD-fed NOX2-FL mice.

To determine if deletion of NOX2 from macrophages can prevent diet-induced memory impairment [30], CD- and HFD-fed NOX2-FL and mNOX2-KO mice were evaluated using the fear conditioning assay as described in Methods. Data show that freezing in response to the tone was significantly decreased in HFD-fed NOX2-FL mice as compared to CD-fed NOX2-FL mice when measured immediately after tone onset, and also when measured 1 and 2 minutes after tone onset (minutes 6, 7,8 of tone test; Fig 4A), suggesting impaired memory of the tone cue. However, HFD-induced impairment was completely prevented in mNOX2-KO mice (Fig 4A).

Analyses of markers of synaptic density and inflammation were measured in the anterior neocortex, a CNS site of increased NOX activity/expression following HFD [13]. Evaluations of synaptic density were based on expression of the post-synaptic marker synapse associated protein 97 (SAP97) and total and phosphorylated forms of the pre-synaptic protein synapsin 1 (SYN1). Quantification of total SYN1 and SAP97 expression revealed no differences in expression between groups (data not shown), but levels of phosphorylated SYN1 were significantly decreased by HFD in NOX2-FL (t_(31)_ = 3.09, p = 0.0042) but not mNOX2-KO mice (Fig 4B). Inflammation was evaluated by measuring the expression the pro-inflammatory/pro-oxidant enzymes inducible nitric oxide synthase (iNOS) and cyclooxygenase 2 (COX2), as well as matrix metalloproteinases MMP2 and MMP9. Data show significant, HFD-induced increases in both iNOS (t_(31)_ = 2.54, p = 0.0162) and MMP2 expression (t_(31)_ = 3.45, p < 0.0016) in NOX2-FL, but not in mNOX2-KO mice (Fig 4B). COX2 and MMP9 were not affected by diet in either strain of mice (data not shown).

## Discussion

Data in this manuscript suggest that NOX2-positive macrophages are involved in the pathophysiology of diet-induced obesity. Specifically, while both NOX2-FL and mNOX2-KO mice became obese following HFD, mNOX2-KO mice had attenuated adipose injury and inflammation, improved glucose tolerance, normalized serum lipids, and preserved cognitive function compared to NOX2-FL mice. These data contribute to the growing body of literature describing the sensitivity of the brain to obesity-induced metabolic dysfunction [31], and the role for NADPH oxidase in neurotoxic brain inflammation [17]. While these data reinforce the link between NADPH oxidase and high fat diet-induced brain injury, the relative contribution of NOX2-based signaling in brain-resident microglia versus peripheral myeloid cells is not clear. Nonetheless, these data are consistent with studies linking increased NADPH oxidase activity/expression to obesity-induced oxidative stress and inflammation [32],[13],[33],[34]. However, it is important to note that NADPH oxidase mediates a variety of *physiologic* processes, including developmental and differentiation pathways, intracellular hormonal signaling, and cognitive function [17],[35]. Furthermore, recent expansion of the genome databases has identified several homologues of NOX2 (NOX1-5, DUOX1-2) which differ in their activation requirements and mediate diverse and pleiotropic actions (reviewed in [36]). Indeed, cell culture and animal data indicate that maintenance of adipocyte function and insulin sensitivity *requires* functional NOX4 activity [37],[38], contraindicating broad-based NAPDH oxidase inhibition in clinical settings. Using novel mice with conditional NOX2 deletion, this manuscript raises the possibility that the pro-inflammatory, detrimental consequences of NADPH oxidase activation in macrophages can be pharmacologically separated from physiologic NADPH oxidase-based signaling in other key cells such as neurons and adipocytes, suggesting that targeted NOX2-based therapies could preserve physiologic function in the context of obesity.

One important pathway through which NOX2 could undermine metabolic function is oxidative stress. NOX2 is the prototypical catalytic subunit of NADPH oxidase, which is a major source of reactive oxygen species in many different cell types [39]. With regards to obesity, data suggest that NADPH-driven oxidative stress in adipose is an early event in the development of metabolic syndrome, triggering increased production of adipokines and elevation of systemic oxidative stress [14]. Oxidative stress has been shown in turn to impair insulin secretion by pancreatic β cells [40], prevent PPARγ activation and translocation [41], and disrupt skeletal muscle glucose transport [42]. While our study focused primarily on visceral adipose, other data implicates NADPH oxidase in vascular dysfunction and insulin resistance [43], and in liver injury/oxidative dysfunction following HFD [44]. Conversely, a recent study by Costford et al suggests that global NOX2 deletion can increase vulnerability to HFD-induced insulin resistance, with knock-out mice showing enhanced hepatic steatosis and inflammation [45]. There are some potentially key experimental differences that might explain the incongruence between data presented in this paper and the comprehensive study by Cosftord et al, including that mice in Costford’s study were started on HFD at weaning rather than as adults. It is also quite likely that increased vulnerability in global NOX2 deficient mice might reflect loss of NOX2 function in non-myeloid cells. While liver injury and inflammation was not directly assessed in the present study, it is noteworthy that HFD-induced hyperlipidemia was nearly completely prevented in mNOX2-KO mice, suggesting preservation of liver homeostasis in these mice.

NADPH oxidase is also integrally involved in pro-inflammatory signaling in macrophages [46], and accumulation of activated macrophages is in adipose thought to be key in translating local changes to systemic inflammation and metabolic impairment [47],[4]. Indeed, the accumulation of macrophages and macrophage-based inflammatory signaling might be what makes visceral obesity so metabolically destabilizing [48]. For example, the number of macrophages has been estimated at two- to four-fold higher in visceral as compared to subcutaneous fat irrespective of adiposity levels [49]. Likewise, pro-inflammatory cytokine expression is elevated in visceral as compared to subcutaneous fat [50], and inflammatory signaling in visceral fat has been repeatedly and directly linked to obesity-related insulin resistance and type 2 diabetes (reviewed in [51]). Increased proinflammatory cytokines can trigger insulin resistance by several mechanisms, including activation of SOCS3 expression [52] and/or the activation of numerous intracellular serine kinases such as jun N-terminal kinase (JNK) and inhibitor of κB kinase (IKK) [53]. Data from our labs and others clearly show the critical role that NADPH oxidase plays in the release of cytokines including TNFα, IL-6, and IL-1β from macrophages [46], [54]. While previous reports suggest that the majority of NOX2-positve cells in adipose are Iba1-positive monocyte/macrophages [15], other myeloid-linage cells within visceral adipose could also contribute to diet-induced changes in adipose inflammation. For instance, data suggest that eosinophils may actually maintain adipose tissue homeostasis during obesity via IL-4 mediated promotion of alternative M2 polarization in tissue macrophages [55]. As the exact role of NOX2 in cytokine release from eosinophils is not known, the possibility exists that NOX2 deletion from eosinophils enhances release of anti-inflammatory M2 cytokines at the expense of pro-inflammatory M1 cytokines, similar to what occurs in macrophages [56]. Collectively, data in this manuscript raise the possibility that inhibition of NOX2 within visceral immune cells may be sufficient to prevent the pattern of visceral inflammation that precipitates obesity-induced metabolic and neurologic decline. This approach to neuroprotection is especially exciting as peripheral immune cells can be more directly and easily manipulated than brain-resident inflammatory pathways, which unfortunately require that the entire body be exposed to the high drug levels needed to breach the blood-brain barrier.

While mNOX2-KO mice were protected from many of the metabolic and neurologic effects of HFD, it is clear that both strains of mice became obese in response to HFD. Obesity affects more than 35% of Americans and contributes to an estimated $147 billion in annual medical costs [7] via ties to type 2 diabetes, cardiovascular disease, and dementia [57],[58]. While the components of a healthy diet are well known, numerous societal (poverty, food deserts, irregular/sedentary work schedules) and physiological (stress, leptin resistance, high metabolic efficiency) factors synergize to hinder weight loss. However, it is significant to note that clinical studies indicate that as many as 30% of obese individuals may have a “healthy obese” phenotype without physiologic dysfunction [59],[60], indicating that metabolic impairment is not caused by obesity *per se*. Furthermore, the key distinction between healthy and unhealthy obesity may indeed be the degree of macrophage-driven inflammation in adipose (reviewed in [61]). For example, macrophage crown-like structures in adipose tissue have been directly linked to insulin resistance [62] and macrophage-associated local inflammatory response is thought to drive other adverse effects of obesity such as adiposopathy [63]. While cause and effect between macrophage reactivity and adipose pathology are not well established, data in this manuscript suggests that macrophage reactivity is a cause rather than a consequence of adiposopathy and mediates, at least in part, the pathologic processes of metabolic and neurologic dysfunction. This scenario is supported by other studies demonstrating that prevention of inflammatory signaling in myeloid cells not only prevents high-fat diet-induced obesity and metabolic dysfunction, but also increases brown adipose tissue activity, white adipose browning, and energy expenditure [64], [65]. While energy expenditure was not measured in the present study, the decreased expression of markers of ER stress in visceral adipose suggests improved adipose physiology. Likewise, as adiposity in mNOX2KO mice was delayed without any obvious changes in energy intake, these collective data indicate at least a temporary increase in overall energy expenditure in mNOX2KO mice. As obesity remains stubbornly prevalent and resistant to clinical remediation [66], these data suggest that new therapies to preserve health in the context of today’s complex environment could be based on manipulation of NOX2 signaling in macrophages.
